# Draft Genome Sequence of *Lactobacillus plantarum* Strain AY01, Isolated from the Raw Material of Fermented Goat Milk Cheese

**DOI:** 10.1128/genomeA.00737-13

**Published:** 2013-10-10

**Authors:** Xiao-Ran Li, Fu-Ming Gong, Hua-Jun Zheng, Zhong-Hua Zhang, Yi-Yong Luo, Chen-Jian Liu

**Affiliations:** Laboratory of Applied Microbiology, Faculty of Life Science and Technology, Kunming University of Science and Technology, Kunming, Chinaa; Chinese National Human Genome Center at Shanghai (CHGC), Shanghai, Chinab

## Abstract

*Lactobacillus plantarum* is an important probiotic that is isolated mostly from fermented foods. Here, we report the first draft genome sequence of *L. plantarum* strain AY01, isolated from the raw material of fermented goat milk cheese. This bacterium, with optimum growth at 30°C, has a G+C content of 43.68%.

## GENOME ANNOUNCEMENT

Fermented goat milk cheese is a unique dairy product in Yunnan Province, China. A local cranberry juice is added to fresh goat milk. After fermentation, cheese is produced. *Lactobacillus plantarum* strain AY01 was isolated from the raw material of fresh goat milk. *L. plantarum* is an important lactic acid bacterial species commonly used as a probiotic.

The nucleotide sequence of the *L. plantarum* strain AY01 genome was determined by using an Illumina HiSeq 2000 platform. A 300-bp paired-end library was constructed and sequenced, producing 24,033,038 pairs of 100-bp reads. One hundred twenty-seven contigs (>500 bp) with a total size of 3,319,865 bp were assembled using Velvet version 1.2.03 (1), providing 723-fold coverage. The average contig length is 26,140 bp and the maximum contig length is 326,866 bp, with the overall G+C content of the *L. plantarum* strain AY01 genome being 43.68%.

A total of 3,174 protein-coding sequences (CDSs) with an average length of 859 bp were identified by combining the prediction results of Glimmer 3.02 (2) and Z curve (3), occupying 82.1% of the genome. Defined biological functions were assigned to 2,434 CDSs (76.7%), and another 597 CDSs (18.8%) are homologous to sequences encoding hypothetical proteins in other organisms, while 143 CDSs (4.5%) have no database match. Among all the predicted proteins, 1,811 proteins (57.1%) were assigned to functional COG categories (4) and another 532 proteins (16.8%) were assigned as having a general function prediction only. In addition, 1,317 genes (41.5%) were involved in 153 different pathways. Those for the Embden-Meyerhof-Parnas (EMP), fatty acid biosynthesis, purine, and pyrimidine metabolism pathways were complete. Fifteen amino acids, not including methionine, valine, leucine, isoleucine, and phenylalanine, could be *de novo* synthesized. The genome contains 66 tRNA genes and 2 rRNA operons.

A comparative analysis of 3,174 proteomes using GenBank found significant matches to the *L. plantarum* genome, with 1,328 proteins (41.8%) matching *L. plantarum* strain WCFS1, 472 proteins (14.9%) matching *L. plantarum* strain JDM1, and 459 proteins (14.5%) matching *L. plantarum* subsp. *plantarum* strain ATCC 14917.

This is the first genome sequence of *L. plantarum* strain AY01, and its availability will provide a better-defined genetic background for future studies on milk fermentation.

### Nucleotide sequence accession number.

The genome shotgun project for *L. plantarum* AY01 has been deposited at GenBank under the accession no. AVAI00000000.
